# Significant improvement of reverse leakage current characteristics of Si-based homoepitaxial InGaN/GaN blue light emitting diodes

**DOI:** 10.1038/s41598-019-38664-x

**Published:** 2019-01-30

**Authors:** Moonsang Lee, Hyun Uk Lee, Keun Man Song, Jaekyun Kim

**Affiliations:** 1 Korea Basic Science Institute, Daejeon, 34133 Republic of Korea; 2 Advanced Nano-surface Research Group, Korea Basic Science Institute, Daejeon, 34133 Republic of Korea; 3 Device Development Department 2, Technology Development Division, 109, Gwanggyo-ro, Yeongtong-gu, Suwon-Si, 9 Gyeonggi-do 16229 Republic of Korea; 4 Department of Photonics and Nanoelectronics, Hanyang University, Ansan, 15588 Republic of Korea

## Abstract

The nature of reverse leakage current characteristics in InGaN/GaN blue light emitting diodes (LEDs) on freestanding GaN crystals detached from a Si substrate is investigated for the first time, using temperature-dependent current-voltage (*T*-*I*-*V*) measurement. It is found that the Si-based homoepitaxial InGaN/GaN LEDs exhibit a significant suppression of the reverse leakage current without any additional processes. Their conduction mechanism can be divided into variable-range hopping and nearest neighbor hopping (NNH) around 360 K, which is enhanced by Poole-Frenkel emission. The analysis of *T*-*I*-*V* curves of the homoepitaxial LEDs yields an activation energy of carriers of 35 meV at −10 V, about 50% higher than that of the conventional ones (E_a_ = 21 meV at −10 V). This suggests that our homoepitaxial InGaN/GaN LEDs bears the high activation energy as well as low threading dislocation density (about 1 × 10^6^/cm^2^), effectively suppressing the reverse leakage current. We expect that this study will shed a light on the high reliability and carrier tunneling characteristics of the homoepitaxial InGaN/GaN blue LEDs produced from a Si substrate and also envision a promising future for their successful adoption by LED community *via* cost-effective homoepitaxial fabrication of LEDs.

## Introduction

Significant development of high luminescence efficiency in InGaN/GaN light emitting diodes (LEDs) has offered new futuristic applications such as automotive headlamps, traffic signals, displays, and general lighting^1–3^. Due to the absence of natural GaN materials, conventional InGaN/GaN LEDs were fabricated on foreign substrates, such as sapphire (Al_2_O_3_), Si, and SiC^4–6^. This, however, generates high dislocation densities (10^8^–10^10^/cm^2^) due to significant lattice mismatch and thermal expansion coefficient difference between substrates and films, thus deteriorating the device performances^7^. Even though InGaN/GaN LEDs using freestanding (FS) GaN wafers have been expected to open a promising way to overcome the degraded device characteristics, the fabrication cost and size limitation of conventional FS-GaN have hindered their successful adoptions in industry^8^.

Recently, we successfully demonstrated homoepitaxial InGaN/GaN blue LEDs on FS-GaN substrate, which was grown and detached from a Si substrate^9^. In spite of using a Si substrate as the support, the InGaN/GaN LEDs showed excellent optoelectronic properties, and a possibility to achieve high device performance with cost-competitiveness and large diameter scale. Although the demonstration of the InGaN/GaN LEDs on FS-GaN extracted from a Si substrate is considered the remarkable implications for new opportunities in the field of InGaN/GaN LEDs, little about their leakage current characteristics has been investigated so far. Since a leakage current determines the device lifetime, luminescence efficiency, and electrostatic discharge sensitivity^10–12^, a great number of attempts has been employed to suppress a reverse leakage current characteristics in InGaN/GaN LEDs using complicated processes such as a design of electron blocking layer (EBL), and surface passivation^13,14^.

In this paper, we performed temperature-dependent reverse leakage current measurement of InGaN/GaN LEDs using FS-GaN extracted from a Si substrate to investigate their transport mechanism in a reverse bias regime and introduce the nature of their remarkable reverse leakage current characteristics, which was achieved without any external processes. We expect that this will provide the LED community with the valuable and promising information regarding the device performances of the homoepitaxial InGaN/GaN LEDs.

## Experimental

**Figure 1:**
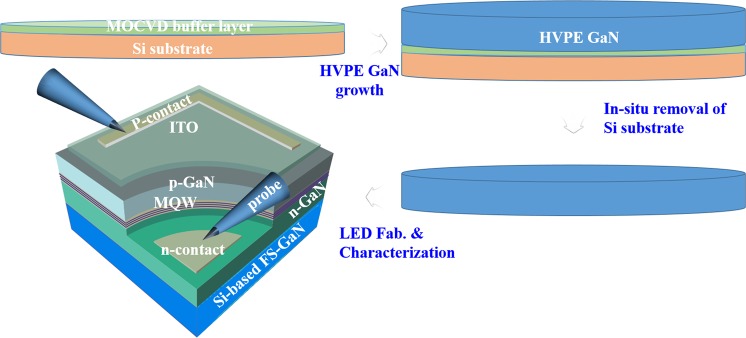
Schematic diagram of the fabrication and characterization procedure of a Si-based homoepitaxial InGaN/GaN LED.

InGaN/GaN multi quantum well (MQW) LEDs with peak emission wavelength of ~440 nm were grown using MOCVD (Aixtron G3 2600) on 2-inch HVPE FS-GaN extracted from a Si substrate (LED I). The device structures and fabrication procedures were detailed in Fig. 1 and ref.^9^. The epitaxial structure comprised a 3.5-µm-thick n-type GaN grown at 1020 °C, 4 pair multi quantum well active layers (MQWs) consisting of InGaN well (3 nm) and GaN barrier (10 nm), grown at 820 °C, and 150 nm-thick p-type GaN grown at 1020 °C. To compare the reverse leakage current characteristics in InGaN/GaN LEDs fabricated using FS-GaN grown from a Si substrate, conventional InGaN/GaN LEDs with corresponding structures and the peak emission wavelength were fabricated on 2-inch Al_2_O_3_ substrate (LED II). All the LED structures were fabricated with conventional lateral chip using conventional photolithography, dry etching, and metallization, which defined 350 µm × 350 µm chip size. Reverse leakage current for both LEDs were measured at reverse voltage of −15 *V* to 0 V in the temperature range of 80–400 K. The defect densities of LED I and LED II were estimated to be 1 × 10^6^ cm^−2^ and 5 × 10^8^ cm^−2^, respectively, which was confirmed by micro photoluminescence (PL) mapping. (Not shown in this paper).

## Results and Discussion

**Figure 2:**
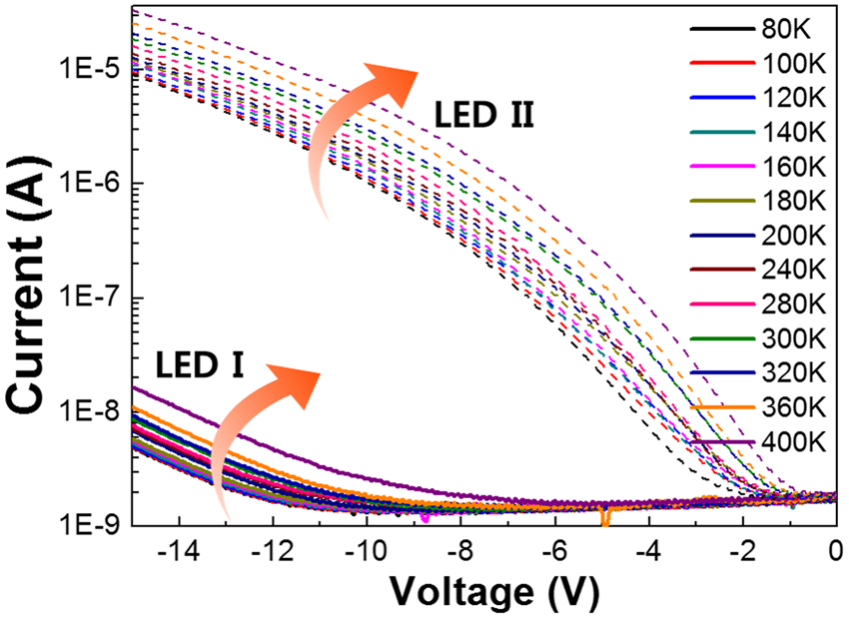
Temperature-dependent current-voltage (*T*-*I*-*V*) characteristics of (**a**) LED I and (**b**) LED II under the reverse bias in the temperature range of 80‒400 K.

Figure 2 shows the representative temperature-dependent current-voltage (*T*-*I*-*V*) characteristics for LED I (solid), and LED II (dash). Regardless of the substrate materials, the reverse currents of LEDs gradually increase with the reverse voltage and temperature. It is worthy to note that the characteristics of reverse leakage current for LED I is superior to that for LED II in all the temperature ranges. The leakage currents for LED I, and LED II at a reverse voltage of −5 *V* are −1.5.0 × 10^−9^ A, and −8.9 × 10^−8^ A at 300 K, respectively. Obviously, a significant suppression of the reverse leakage current in LED I was observed, compared to that of LED II. In addition, it is essential to note that the reverse leakage current in LED I is reduced more than one or two orders of magnitude with respect to the other results using additional leakage current suppression processes^15–17^. We believe that this is attributed to the reduced dislocation density in LED I. One can clearly observe that the behaviors of the reverse leakage current curves for LED I and LED II appear quite similar in a way that their current under the reverse bias increases sub-linearly. However, their dependence on the temperature and the magnitude of reverse bias demands further detailed investigation. Based on *T*-*I*-*V* measurement results, it can be assumed that they have the same transport mechanisms with different onset points in terms of the temperature and applied reverse bias. It was already reported that the characteristics of the reverse leakage current for both LEDs takes the applied voltage and temperature into the account, suggesting the combination of tunneling current and thermal generation current. At low applied reverse bias, the slope of the reverse *I*–*V* curves are significantly dependent upon the temperature, indicative of the presence of thermally activated process^18^. It is well known that a tunneling through defect sites dominates the conduction mechanism in this regime^19^. A tunneling in LED I does not seem to dominate by the reverse bias of 8 *V*, where an abrupt increase in the current occurs. On the contrary, the reverse leakage current in LED II starts to increase at the reverse voltage of about 1 V. Note that the temperature- and field-dependence on the reverse leakage current in LED II appears relatively more severe, compared to that in LED I. We speculate that this is attributed to higher dislocation density in LED II, which can stimulate the field-dependent carrier tunneling at the elevated temperature.

**Figure 3:**
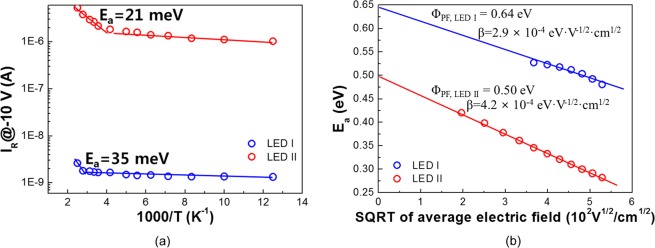
(**a**) Arrhenius plot of the reverse leakage current of LED I and LED II as a function of reciprocal temperature, measured at −10 *V*. (**b**) Thermal activation energies (*E*_*a*_) of LED I and LED II from Arrhenius plots as a function of the square root of the average internal electric field. SQRT implies the square root.

In order to get further insight on the transport mechanism of the reverse leakage current in the InGaN/GaN LEDs, we conducted Arrhenius plots of the reverse leakage current as a function of reciprocal temperature under the reverse bias of −10 *V* for LED I and LED II, as illustrated in Fig. 3. As seen in the figure, two distinct scaling behaviors are clearly found at low and high temperature regimes in both LED I and LED. It is well known that the drift-diffusion current and Sah-Noyce-Shockley generation-recombination current under reverse bias can be neglected in wide-bandgap GaN-based LEDs^20^. Hence, main transport mechanisms could be attributed to variable-range hopping (VRH) conduction mechanism and field-enhanced thermionic emission, also known as Poole-Frenkel emission, with different slope regimes^20^. In region below 320 K, the reverse leakage current in both LEDs appears to be slightly dependent upon the temperature. This implies that the VRH conduction mechanism is responsible for the leakage tunneling characteristics, where the carriers hop from the valence band of p-GaN to the conduction band of n-type GaN *via* localized deep trap levels^21,22^. The relationship between current and driving voltage for VRH can be given by^23^:

1 $${\rm{I}}\propto {I}_{0}\,\exp \,\{-{(\frac{{T}_{0}}{T})}^{\frac{1}{4}}\}$$

where *T*_0_ is the characteristic temperature.

From the fitting (1) onto Fig. 3, LED I and LED II follow a VRH conduction below the temperature of 360 K and 280 K, respectively. VRH conduction mechanism does not fit well anymore over 360 K and 280 K for LED I and LED II, respectively. It is well established in the nearest neighbor hopping (NNH) model that the transport of electrons from valence band of p-GaN to the conduction band of n-GaN occurred through the multistep hopping across the close states in space but far ones in energy at high temperature. This can be enhanced by the Poole-Frenkel emission^24^. The leakage current increases exponentially as a function of 1/T. The slope indicates the thermal activation energy *E*_*a*_, implying the energy for those trapped carriers in deep trap levels to escape^25^. This can be explained by the following equations^20,26,27^.

2 $${\rm{I}}\propto \exp \,(-\frac{{E}_{a}}{kT})$$

3 $${E}_{a}={{\rm{\Phi }}}_{PF}-{\beta }_{PF}{F}^{1/2}$$

where *E*_*a*_ is the thermal activation energy, *k* is the Boltzmannn constant, *Φ*_*PF*_ is the Poole-Frenkel barrier height of carriers trapped in deep trap centers without external electric field, corresponding to extrapolation of *E*_*a*_ at the bias of *V* = 0 *V*, *β*_*BF*_ is the Poole-Frenkel constant (theoretical value in GaN: 3.28 × 10^−4^ eV·V^−1/2^·cm^1/2^), and *F* is the local electric field strength exerting on the deep centers. The Poole–Frenkel constant accounts for how the average barrier height of deep centers interacts with the external bias^23^. Linear fittings by eq. (2) confirm thermal activation energies of 35 meV, and 21 meV for LED I, and LED II, respectively. One can notice that the activation energy for LED I is much higher than that for LED II. We assign that this ascribes lower dislocation density in LED I, notwithstanding the use of a Si substrate as a supporting material. This is in good agreement with the reported results that higher dislocation causes lower thermal activation energy^23,28^. Furthermore, *Φ*_*PF*_ can be extracted to be 0.64 eV, and 0.50 eV for LED I, and LED II from the extrapolation of the thermal activation energy as a function of average internal electric field, *F*^*1/2*^, respectively, as depicted in Fig. 3(b). The average internal electric field can be estimated by *F* = (*V*_*bi*_ − *V*)/*d*, where *V*_*bi*_ is the built-in voltage of the InGaN LEDs, *d* is the depletion width^29^. Capacitance-voltage (C-V) measurements were conducted to compute the internal electric field. (Not shown) We can easily notice that the *E*_*a*_ decreases with the increase in the reverse bias. This indicates carriers can escape from localized deep centers more easily at higher reverse voltage, implying participation of escaped carriers into leakage current. Notably, the value of *Φ*_*PF*_ for LED I is much higher at any bias than that for LED II. We speculate that the low threading dislocation density of the homoepitaxial LED I contribute to the enhancement of *Φ*_*BF*_. Note that the Poole-Frenkel constant of LED I exhibits on the order of 10^−4^ eV·V^−1/2^·cm^1/2^. It is evident that the electrical characteristics of defects in LED I using a Si substrate as a support seems quite similar to one in LED II using sapphire substrates.

**Figure 4:**
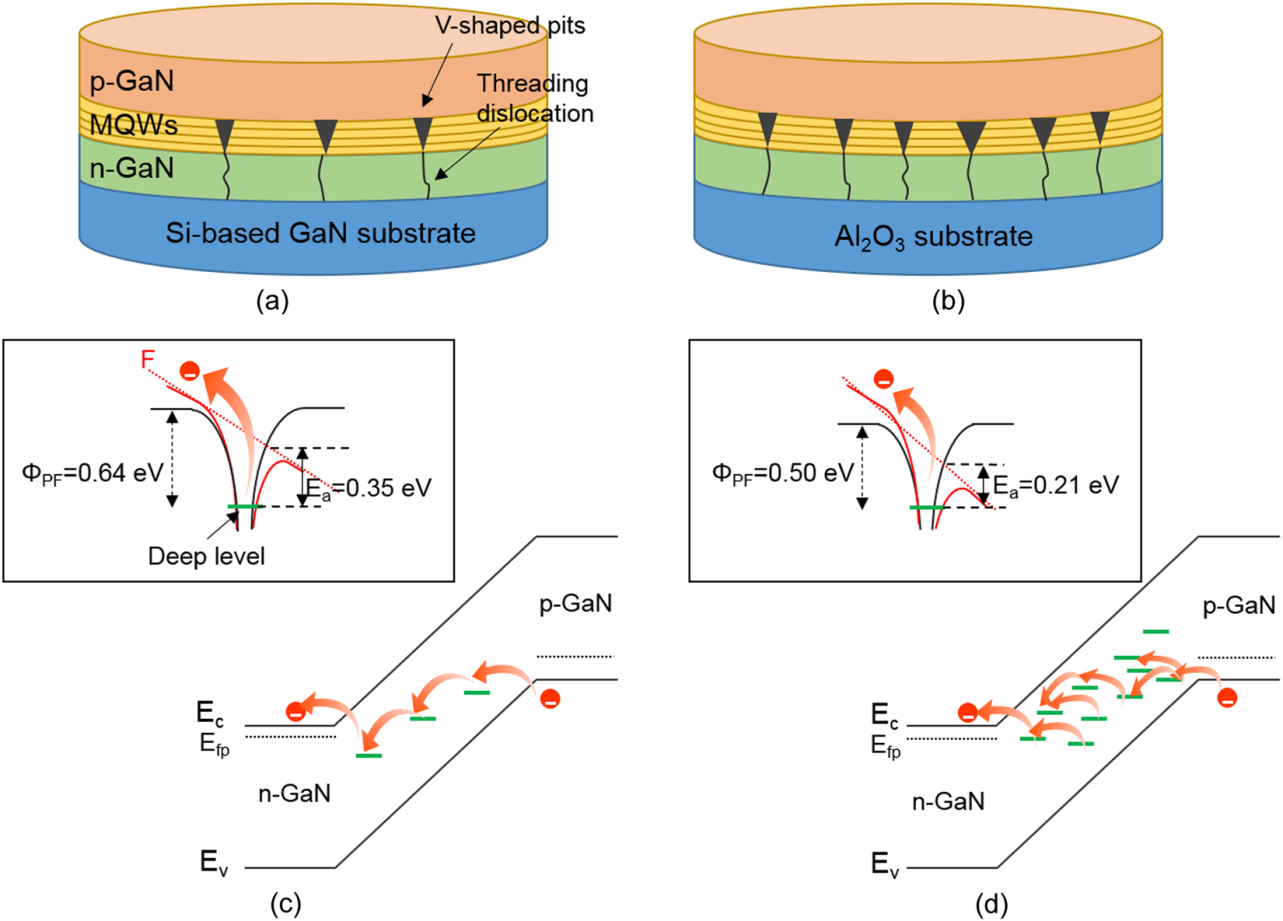
Schematic diagrams of the device structures for (**a**) LED I, and LED II. (**c**,**d**) Illustrate the charge conduction for LED I, and LED II, respectively. The insets depict the schematic diagrams of the Poole-Frenkel effect for LED I, and LED, respectively.

To visually describe and compare the transport mechanisms of two different LEDs used in this study, we illustrate the schematics of LED structure with different defect density and energy diagrams of the p-i-n junction under reverse bias, as shown in Fig. 4(a,b). As mentioned above, conventional InGaN/GaN LEDs on sapphire substrates bear relatively higher number of threading dislocation density, often resulting in V-shaped pits embedded in InGaN/GaN multiple quantum wells (MQWs). However, the threading dislocation density of LED I is significantly reduced due to the nature of homoepitaxial growth, consequently leading to the reduced density of V-shaped defects in MQW layers as well. Although V-shaped defects exhibit other advantageous effect, it is well known that a threading dislocation behaves as the leakage current path, thus deteriorating the reverse leakage current characteristics of InGaN/GaN LEDs^30,31^. Figure 4(c,d) illustrate the schematic diagram of the conduction mechanism of LED I and LED II based on an origin of reverse leakage current under the reverse bias, respectively. These figures schematically describes relative higher activation energy of trapped carriers at deep centers for the conduction in homoepitaxial InGaN/GaN LED, compared to one of LED II. One can expect that higher activation energy of trapped carriers in LEDs, lower chance of carriers escaping from the deep traps and also lower leakage current under the reverse bias. Not surprisingly, the magnitude of reverse leakage current in InGaN/GaN LEDs is also associated with the density of threading dislocations in LEDs. As a result, LED II possesses more than 3 orders higher reverse leakage current magnitude, compared to that of LED I over wide range of temperature. This is in good agreement with the recent literature^32^. Additionally, a reduced density of deep trap levels in the homoepitaxial InGaN/GaN LEDs assist a decrease in a tunneling probability of carriers into the conduction band edge of the p-GaN layers. Besides, the reverse current distribution of LED I in a wafer exhibit much lower values, compared to that of LED II since LED I exhibits much lower dislocation density^33^.

## Conclusions

We report the reverse leakage characteristics of InGaN/GaN LEDs on FS-GaN peeled off from a Si substrate for the first time, using *T*-*I*-*V* measurement. Compared to the reverse leakage current characteristics of conventional LEDs, the Si-based homoepitaxial LED shows significantly suppressed one without any supplementary processes. Additionally, the transport mechanism of the homoepitaxial InGaN/GaN blue LEDs is dominated by VRH and Poole-Frenkel emission, which is similar to that of conventional blue LEDs. The lower reverse leakage current of the homoepitaxial InGaN/GaN LEDs is attributed to a higher thermal activation energy (E_a_ = 35 meV@ −10 V) and a reduced dislocation density. This study offers strong support for the viability of InGaN/GaN LED on FS-GaN peeled off from a Si substrate to achieve the highly reliable homoepitaxial InGaN/GaN LEDs with high cost-effectiveness and large diameter-scale.
